# Remedies and Their Uses

**Published:** 1906-11-24

**Authors:** 


### Remedies and their Uses.

### Validol.

This is an ester of valerianic acid and menthol, and is sold either in an effervescing or a non-effervescing form. It contains 30 per cent. menthol, and is a colourless fluid, with a faint pleasant odour and refreshing taste. Externally it has been used as an antipruritic in the form of a 10 to 15 per cent. ointment, or mixed with 10 per cent. camphor (validolum camphoratum) as an analgesic for carious teeth. Internally it is employed as a substitute for valerian root. Kerner (*Klin. Therap. Wochenschr.*, 1903, No. 29) and others have employed it also in cases of epilepsy, in which they consider it possesses certain advantages over bromides. It is slightly stomachic, and is generally easily taken by the mouth. In migraine, gastralgia, and hysterical headache it has been successful, and the internal treatment may be reinforced by its external application over the seat of pain. It is usually given in doses of ten drops thrice daily on a lump of sugar. Kœpke (*Therap. Monartsh.*, 1904, No. 7) has found validol very useful in sea-sickness, ten to fifteen drops being taken when the first symptoms set in. Its general action seems similar to that of valerian; it raises the blood-pressure and is a powerful carminative. It is also a local antiseptic, and may be inhaled in conditions of laryngeal and bronchial catarrh; five drops in a tumbler of water forms an efficient antiseptic mouth-wash (Isaac, *Berl. Klin. Woch.*, 1903, No. 5). It is prepared by Zimmer and Company.

